# Qualitative Study of Community Pharmacists’ and General Practitioners’ Views toward Pharmacovigilance in Lithuania

**DOI:** 10.3390/healthcare9081072

**Published:** 2021-08-20

**Authors:** Agne Valinciute-Jankauskiene, Loreta Kubiliene

**Affiliations:** Department of Drug Technology and Social Pharmacy, Lithuanian University of Health Sciences, Sukileliu Ave. 13, LT-50166 Kaunas, Lithuania; loreta.kubiliene@lsmu.lt

**Keywords:** adverse drug reaction, general practitioners, community pharmacists, reporting, pharmacovigilance

## Abstract

Lithuania is the leader in pharmacovigilance among the three Baltic countries. However, comparisons with other European countries are difficult because the reported number of adverse drug reactions (ADRs) in Lithuania is too low to rely on in terms of the annual use of medicines by the population over the year. The aim of this study was to explore challenges related to the understanding and practices of general practitioners and community pharmacists in reporting ADRs in Lithuania. The qualitative study approach of face-to-face interviews was used. All interviews were recorded, transcribed verbatim, and thematically analyzed. Twelve interviews with general practitioners and community pharmacists were conducted from March 2020 through December 2020. All participants had a basic knowledge of pharmacovigilance, but only four participants reported ADRs to the interviewer. Six main barriers regarding ADR reporting were identified, and appropriate interventions were suggested. The importance of collaboration between physicians and pharmacists was highlighted, and the need for guidelines supporting collaboration was expressed. Medications are becoming more complex, and comprehensive medication management is key for the optimization of patient outcomes. Our results reveal the need to improve and innovate the current pharmacovigilance system at all levels, starting from education for pharmacy and healthcare students and continuing through the development of ADR procedures.

## 1. Introduction

An adverse drug reaction (ADR) is defined by the European Medicine Agency as “A response to a medicinal product that is noxious and unintended.” This definition covers any ADR following the use of medicine, noxious and unintended effects resulting in medication errors, and uses outside the terms of the marketing authorization, including the misuse and abuse of the medicinal product [1]. All medicines have potential side effects, which unfortunately are an important cause of patient morbidity and mortality [2]. ADRs have substantial economic as well as health costs, because they frequently lead to hospital admission, hospitalization prolongation, and emergency room visits [3]. In Europe and the United States, ADRs are among the top ten causes of death. According to various data sources, between 0.7% and 6.5% of adult patients are hospitalized for ADRs [4,5,6]. Children also experience ADRs when treated with medications. It has been shown that up to two in ten hospitalized children experience adverse reactions [7].

A large change in the system of pharmacovigilance occurred following the thalidomide tragedy in the 1960s. Spontaneous reporting systems (SRSs) were established and became systematic, organized, and regulated [8]. The SRSs enabled the early detection of new, rare, and serious ADRs by cost-effectively covering the entire population [9]. Pharmacovigilance plays a critical role in medical safety, making the continual growth and improvement of SRSs essential to ensure the safety of all medicines by continuous monitoring throughout their use in healthcare practice [10]. However, the effectiveness and efficiency of pharmacovigilance depend on the active participation of all stakeholders, namely, pharmaceutical companies, government drug regulation agencies, healthcare professionals (HCPs), and patients. The estimation that only 6% of all ADRs are reported highlights the existing weakness known as under-reporting [11].

The State Medicines Control Agency of Lithuania (SMCA), established in 1995, created a legal basis on which to regulate national pharmacovigilance. Since 2001, when a form of a report for observed ADRs was approved by the SMCA, ADR reporting has been obligatory for HCPs [12]. Statistics provided by the SMCA show that the number of submitted ADR reports from HCPs and pharmacists has not changed significantly over the past 20 years. These statistics show that the least impact on pharmacovigilance in Lithuania was found among community pharmacists (CPs), who were in last place in ADR reporting [13].

Many studies have been conducted to investigate the knowledge of, perception of, and barriers to ADR reporting by CPs and HCPs worldwide [14,15,16]. However, very little is known about factors that influence ADR reporting by CPs and HCPs in Lithuania. Only one reason was identified in a study of general practitioners (GPs) more than ten years ago, namely, a lack of time [17]. The minimal and outdated evidence on ADR reporting highlights the importance of updating the existing evidence base.

According to our knowledge, this is the first qualitative study to explore and understand the experiences, perceptions, and possible barriers of ADR reporting for GPs and CPs in Lithuania that could lay the foundation for broader investigation in the future.

## 2. Materials and Methods

### 2.1. Ethical Approval

The study (reference no. BE-2-59) was approved by the Kaunas Regional Biomedical Research Ethics Committee, Lithuania. All participants provided signed informed consent.

### 2.2. Study Design

A qualitative research approach was selected to perform this study, because this method enables the identification of gaps that remain unnoticed by survey-based research methods [18]. Rather than focusing on numerical data, we sought to better understand the issues that face study participants and to explain their behavior by analyzing their thoughts and feelings [19]. A semi-structured interview guide was developed based on a literature review, previously performed studies, and common pharmacovigilance practices in Lithuania [11,15,17,20,21].

### 2.3. Study Sampling and Recruitment

Several sampling strategies can be used in qualitative research. Although the required sample size was not determined, data collection should continue until data saturation is reached [22]. Therefore, the number of participants depends on the richness of the data. Nonetheless, Miles and Huberman [23] proposed that more than 15 cases could make analysis difficult and complicated. The GPs and CPs were recruited by a combination of purposive and convenience sampling through researchers’ contacts. The targeted participants were recruited from public and private hospitals and different network pharmacies, taking their work experience into account. All GPs and CPs voluntarily participated in this study.

### 2.4. Data Collection

The interviews were carried out from March 2020 through December 2020. Written informed consent was obtained from each of the GPs and CPs before participating in an interview. Every participant was informed that the interview would be recorded.

All interviews were conducted by the researcher (first author), and the approximate interview duration was 20–45 min. Participants were interviewed until saturation was reached; therefore, no new interviews were conducted after 12 interviews. No new themes emerged after ten interviews, but two more interviews were conducted to conclude that saturation was achieved.

### 2.5. Data Analysis

All interviews were audio-recorded, transcribed verbatim, and thematically analyzed according to a previously described method [24]. The first step was to become familiar with the data collected. Subsequently, the transcripts were arranged and organized in a systematic and meaningful form. This preparatory first step allowed the researcher to identify and generate initial codes from the transcripts, which could later be used to derive themes. All the large data collections were reduced to smaller, relevant, and appropriate text called codes. These identifying codes were openly colored to bring clarity to the entire coding process. In this way, the codes identified would be relevant to the study questions. In other words, only the relevant themes or codes were condensed from the transcripts. The responses were color-coded by re-reading the transcripts, highlighting the relevant text, and matching the codes. There were no pre-set codes, and every code was developed and modified when analyzing the data.

## 3. Results

### 3.1. Demographic Characteristics of Participants

A total of twelve individuals, six GPs and six CPs, were interviewed. Each group consisted of two males and four females. Five participants had ten to twenty years of experience; three participants had up to five years of experience; three participants had five to ten years of experience; and one participant had more than twenty years of experience. The least experienced GP had three months of experience, whereas the least experienced CP had one-and-a-half years. The demographic distribution of the participants is shown in Table 1.

### 3.2. Thematic Analysis of Content

During the thematic content analysis, six major themes and ten subthemes were identified. The themes and subthemes are listed in Table 2.

#### 3.2.1. Theme 1: General Knowledge about the Definitions of Pharmacovigilance and ADR

The participants were asked about their familiarity with the definitions of pharmacovigilance. The responses were as follows:

“This concept to me is usually associated with adverse drug reactions, but also to others. If a pharmacist notices some discrepancies, I think that could also be attributed to the concept.” (CP4)

“It is probably necessary to notice the most adverse reactions there are to the drug and the action and whether there is sufficient action. This is the main pharmacovigilance. This is probably mostly due to adverse drug reaction fixation and its, let’s say, avoidance.” (GP4)

“So pharmacovigilance is what regulates those processes, the safety of drugs, the effectiveness of drugs, and relates to the day-to- day activities where they can mediate between them.” (CP3)

The definition of adverse drug reaction was described as follows:

“An adverse reaction is one that a person does not want from taking that medicine.” (CP4)

“It’s an unwanted reaction, as I understand it, a reaction from the body that just, let’s say, well, isn’t what it should be.” (CP2)

#### 3.2.2. Theme 2: Updating Knowledge on Pharmacovigilance

The participants were asked to assess the knowledge and awareness of their colleagues in pharmacovigilance. The assessment varied between poor, basic, and average; one GP was evaluated as good. During the interviews, several sources of knowledge were identified.

“I read the leaflets from time to time.” (CP4)

“There are trainings, lectures, seminars, online trainings.” (CP1)

“Such courses now, you know, are not very available. Just looking for information and gadgets of all kinds for health professionals.” (GP2)

The GPs were also asked about their knowledge of the CPs and vice versa. The GPs and CPs evaluated the knowledge of the other group better than the group to which they belonged.

“Well maybe I think doctors.” (CP5)

#### 3.2.3. Theme 3: Practices Related to the ADR Reporting

The participants were asked about their practices related to ADR reporting. Mixed practice was observed.

“If I had heard or read about it, that there’s even a kind of system of pharmacovigilance, I’d answer ‘no’” (GP3)

“Theoretically, I know where to go, where to find that form, how to fill it out, and so on.” (CP3)

“I didn’t actually even check that form completely, but I think the patient’s consent is needed.” (CP4)

#### 3.2.4. Theme 4: Barriers to ADR Reporting

The CPs and GPs were asked to express the main barriers toward ADR reporting. Lack of information and education, time, trust, feedback, workload, complexity, limitations of the e-health system, and organizational culture were mentioned.

##### Subtheme 1: Lack of Information and Education

The majority of the GPs and CPs identified a lack of information and education as one of the major barriers for ADR reporting:

“You may need more information to remind the doctor, the same pharmacist, the same patient, let’s say. Somewhat simpler information would make it more accessible. To make them understand, to know that they can apply it and say, ‘So here’s a side effect for me, a side effect. So what can you do?’ It’s really a lack of information. Too little information.” (CP1)

“Yes, in fact, that kind of opinion is probably not very clear (ADR reporting system), because maybe there is a lack of information.” (GP1)

##### Subtheme 2: Heavy Workload

Some of the interviewed participants indicated that a heavy workload created a barrier for reporting:

“This would probably be one of the main reasons why, because it is necessary. We are burdened with all kinds of work and, in fact, it is usually easier to remove and stop prescribing that medicine than to report and record side effects.” (GP4)

##### Subtheme 3: Complicated ADR Reporting Procedure

The complexity of the reporting system was mentioned as another barrier that causes hindrance in the smooth reporting of ADRs. The views were expressed as follows:

“You see, I know about pharmacovigilance, but there’s such a cluttered system. There’s everything needed to fill out, and it takes time. I just haven’t used it and I don’t know if I’ll ever be able to use it.” (GP2)

“Because now all those reports are so complicated that we don’t even fill them out.” (GP3)

##### Subtheme 4: Limitations of the E-Health System

The majority of the participants identified the limitations of the e-health system as a major barrier to reporting. The information in the system about each patient is either incomplete or provided in a complicated way:

“You see a huge list of drugs that were prescribed there, and you end up not knowing what that person is taking today. It should show in the electronic system what is being reimbursed, well, what medications are being prescribed to the patient, and just the whole box that is relevant to this day, not half a year ago. I was looking for the drugs a patient was taking yesterday. It throws me out year 2016 and what he was taking. Then I have to scroll back to this year.” (GP3)

“Especially if you’re taking seven to eight medications, then until you discover who’s there with whom, what doses, especially if you need to adjust something or add something new, it really takes a lot of technical work.” (GP6)

“I’ve really encountered cases like this when we don’t know what the patient is taking. And unfortunately, sometimes when complications and serious side effects develop, only then do you find out.” (GP2)

“Because, for example, we don’t see what the psychiatrist prescribed.” (GP6)

##### Subtheme 5: Organizational Culture

Participants shared their experience with organizational culture regarding ADR reporting:

“We are not even trained. Let’s say we need to fill in some documents here and submit them somewhere. It’s just been talked about in general, maybe from university knowledge, but no one has emphasized that in the work itself.” (CP2)

##### Subtheme 6: Lack of Trust in Authority

During the interview, it was admitted that there was a lack of trust in the authority responsible for ADR collection:

“I think that in other countries the work on drug safety is much fairer, although I do not know. I have the impression that other countries register those things (ADRs) or the system is simplified. I hope that it works much better elsewhere than in Lithuania, and since the Lithuanian market is very small, it does not alter much, let’s say, the statistics at all.” (GP4)

#### 3.2.5. Theme 5: Facilitators of ADR Reporting

##### Subtheme 1: Improvement of the System

During the interview, all participants emphasized that the improvement of reporting procedures and systems would serve as a motivational factor to report ADR:

“I would think it would be best to have some kind of system that you would see, let’s say doctors would see the incompatibility. They would see what drugs they (consumers) buy there, and see more information from other doctors. Because often when talking to patients, usually one doctor prescribes one medication and another doctor has prescribed other medications. If there are still simple (paper-based) prescriptions, it is the case that they (consumers) take three painkillers and they don’t know exactly where they are from, why and how long they take them.” (CP3)

“And I think, in fact, that maybe it’s better. A simpler system would be even faster, well, let’s say, so during work, and you don’t have to sit there after work and fill in those applications. It’s just fast. You open and somehow you quickly gather all the information and it immediately goes there.” (CP1)

“It would be best to somehow simply indicate on the electronic card that there is an adverse reaction before discontinuing or changing the medication. And the questionnaire would be completed automatically.” (GP4)

##### Subtheme 2: Active Support

The interviewed physicians indicated that active involvement of the authorization holders into the ADR reporting procedure is motivating, and that similar activities shown from SMCA would also be motivating:

“Pharmaceutical companies are now promptly electronically notifying and warning of any confirmed side effects of such and such medications.” (GP2)

“I would welcome the fact that, let’s say, if it concerns a manufacturer, you are contacting the manufacturer directly. There needs to be fast communication. If there is a representative, a quick response, arriving by himself, and really providing information, so we have good feedback from manufacturers. And regarding the SMCA webpage, you just have to fill it in (form); that’s all. Nobody will probably eliminate bureaucracy here, probably never.” (GP5)

##### Subtheme 3: ADR Map

During the interview, it was suggested that an additional tool that focuses on groups of ADRs or the most often seen ADRs would better enable them to recognize and learn about drug reactions:

“I think it would be best to do this through training, systematically; to make it more reminiscent of a lecture at a university, because now that we have training, it’s about a specific drug. And here, perhaps, we should look more closely at the other side and distinguish more about those syndromes caused by groups of drugs.” (CP4)

#### 3.2.6. Theme 6: Collaboration between GPs and CPs

##### Subtheme 1: The Importance of Cooperation

When asked about the collaboration between physicians and pharmacists, it was agreed that working together could improve patient care:

“It would be very helpful and great.” (GP3)

“Of course, it’s a team thing” (GP5)

##### Subtheme 2: The Need for a Clear Collaboration Algorithm

Although the participants agreed that collaboration practice is very import, it was also emphasized that there were no clear guidelines or algorithms for practicing such collaboration:

“The essential thing I can say is clarity. It means everyone needs to know who is doing what on that team. If the GP knows that algorithm, how he has to report, how he has to communicate with that pharmacist, then he will communicate; pharmacists in exactly the same way. If he has an algorithm, in which case and when he has to call or write that doctor, then that collaboration will take place. But if you are told to just cooperate, then there will be nothing.” (GP3)

“I would think there should be some kind of system that connects both pharmacists and doctors.” (CP3)

## 4. Discussion

To the best of our knowledge, this is the first qualitative study that explores ADR reporting awareness and practices of GPs and CPs. Even though drug safety and reporting of ADRs are the responsibility of all healthcare providers, pharmacists, and patients, statistics show that marketing authorization holders are the leading reporters in Lithuania [13]. A number of studies have analyzed under-reporting problems among healthcare providers and pharmacists in different countries [14,15,16]. This is the first study that sought to better understand the under-reporting issues in Lithuania and identify the gaps that may remain unnoticed by survey-based research methods [25].

In general, it was found that the majority of GPs and CPs have at least basic knowledge about ADRs and pharmacovigilance activities and know the importance of medication safety. However, a gap in the knowledge of ADR reporting procedures was also found. These results are consistent with similar studies and show a lack of education and unfamiliarity with the guidelines [9]. Notably, not all participants were aware of the pharmacovigilance system in Lithuania. Interestingly, both GPs and CPs rated the knowledge of the other group better than their own.

To determine the sources of ADR information used by the respondents, questions regarding the education in the field of pharmacovigilance were asked. Several sources were mentioned in the participants’ responses, but the authority responsible for pharmacovigilance activities was not among their responses. Participants who had been educated in pharmacovigilance stated that they received such education at university, from the internet, and from marketing authorization holders. All the participants actively took part in various conferences and lectures. However, even though the participants received information about ADRs, in most cases, the information was presented about ADRs of a specific medicine and not about pharmacovigilance in general.

The interviews revealed that CPs mostly rely on medicine compatibility programs installed in pharmacies and their knowledge, whereas GPs rely on information and support provided by marketing authorization holders. This finding is in line with other studies which have shown pharmacy students’ superior pharmacovigilance knowledge compared with medical students [26]. All participants interviewed had detected an ADR once in their professional practices, indicating that both groups were capable of identifying ADRs in their work setting. However, the younger CPs did not tend to report without senior pharmacist approval or clear guidelines, and younger GPs lacked confidence in recognizing ADRs and practical education in ADR reporting procedures. These findings may explain why CPs most likely refer their patients to medical attention when an ADR is detected, and GPs seek help and support from marketing authorization holders who report ADRs to the competent authority [27]. These specific identified behaviors of CPs and GPs need to be addressed when interventions are applied to improve the reporting of ADRs.

During the interviews, participants identified a number of barriers to ADR reporting. As in prior research [9,28], the most discussed barrier in this study was the lack of information and education. The participants had low levels of knowledge about concepts and processes related to pharmacovigilance. This barrier could be targeted by educational interventions through theoretical and practical sessions. In order to avoid short-lived improvement, the educational intervention should be long-lasting, innovative, and engaging. All pharmacists and physicians receive educational training at universities; however, the training is episodic and primarily based on theory. In addition, local reporting guidelines and policies change over time; thus, previous education, especially that received decades ago, is not sufficient in today’s situations.

The other common barrier identified was a heavy workload, which has been known for over a decade [17], and is consistent with the results of other studies [28,29]. Increasing competition between private clinics and efforts to attract as many patients as possible results in increased workloads, higher stress, and limited decision-making. Most working hours involve paperwork. Improvement of time management skills, support from establishing a drug safety department, and additional staff may be comprehensive interventions to alleviate this barrier.

A weak e-health system containing the records of prescribed medications and trust in authority were found to be other contributors impeding the smooth reporting of ADRs by GPs. The limitations named by GPs included the inability to see all medications prescribed to the patient (especially psychotropic medicines), a complicated listing of the prescribed medicines, and lack of additional functions, such as drug compatibility. These problems could be solved by implementing user-friendly updates to the e-health system and a computerized documentation system. The possibility of reporting ADRs by importing data directly from hospitals’ electronic patient records to the ADR form was a priority mentioned by the study participants. Results of previous studies show that the inclusion of hyperlinks on computer desktops and in electronic patient records to online ADR reporting forms is an easy and cost-effective way to change the behavior of health professionals toward spontaneous ADR reports. For example, an ecological study in Portugal using such technology showed that the number of ADR reports increased more than twofold [30]. The participants of this study mentioned that the simple transfer of patients’ data records concerning ADRs directly to a reporting form would increase their likelihood to report ADRs.

Trust in a competent authority responsible for pharmacovigilance activities was another barrier named by GPs. The current authority is seen as an institution purposefully not performing its functions, or operating for the benefit of non-patients. This described barrier does not fall among eight opinions related to the causes of under-reporting and is novel; we did not find a similar outcome in other studies [31]. To overcome this obstacle, interventions, such as the provision of active feedback and support from an authority, initiation of open discussions with healthcare providers, and educational sessions are needed.

An interesting barrier named by CPs toward ADR reporting was *organizational culture*. The results show that reporting ADR was influenced by the culture of reporting in the pharmacy. A culture of reporting ADRs has not been established in pharmacies, making it difficult for CPs to overcome the difficulties of reporting. The interviewed GPs tended to discuss adverse reactions with their colleagues. In contrast, the CPs followed the job description and standard operating procedures of the pharmacy and were most likely to refer the patient to a healthcare provider instead of reporting ADR to the responsible institution. A similar barrier was previously found among nurses, who were socially influenced by the reporting culture in the hospital [28]. An appropriate intervention for these cultural issues may be to organize the cultural influences, targeting the pharmacies, and providing education in the form of workshops to improve reporting.

The spontaneous reporting system depends on the voluntary submission of ADR reports; therefore, the reporting needs continuous stimulation. A recent scoping review showed that the greatest impact was achieved by educational interventions, including theoretical and practical sessions, intensive monitoring systems, and computerized registration [32]. It was also demonstrated that applying multiple interventions has a higher impact than single interventions. Although Lithuania is leading in three Baltic countries, there is room for growth in the reporting of ADRs [33,34]. Considering the identified barriers found in this study, we suggest several interventions (see Table 3) that could be most effective in removing these barriers and improving Lithuania’s current pharmacovigilance situation.

During the interviews, the researchers identified few facilitators who were agreeable to increasing their ability and willingness to report. In addition to the previously mentioned computerized documentation system and active support, the ADR map was highlighted. Similar to the ADR Trigger Tool for monitoring and reporting ADRs developed by the Institute for Healthcare Improvement in 2003, an alternative and simplified method could be suggested [33,35]. The list of trigger medicines, common ADRs, and interactions with other medicines should be offered to CPs and GPs. The tool would allow the identification of recurring risks and contribute to risk reduction strategies for medication safety.

The GPs and CPs who participated in this study agreed that a collaborative practice toward ADR identification and prevention could improve patient outcomes. Participants indicated that they had limited experience working collaboratively, which is consistent with findings from other studies [36]. Both groups expressed a desire to improve communication; however, there are a lack of clear guidelines for how collaboration between GPs and CPs should occur. Guidelines of what information in which situations should be shared between studied groups would facilitate medication-related issues and accelerate their identification.

One possible limitation of our study is the sampling strategies and the sample size. Even though two sampling techniques, purposive and convenience, were used, there is a danger that the results of such research can only be generalized to the subpopulations with the characteristics that define and limit the convenience and purposive samples. The participation of a more significant number and greater diversity of CPs and GPs would increase the value of this study. However, the qualitative data were not mathematically evaluated but relied on opinion and judgment, and the frequencies of occurrence are rarely important. Despite the limited sample size, saturation of the collected data was reached. Future research is planned to address other factors that can dictate how quickly or slowly saturation is achieved in a qualitative study. Although these limits influence the generalizability of results, this study provides valuable insights to improve the understanding of the pharmacovigilance system among CPs and GPs.

## 5. Conclusions

The results of this study show that CPs and GPs had a general knowledge about medication safety; however, their knowledge about the ADR reporting system was either low-level or nonexistent. The main barriers identified in this study were related to a lack of information and education, heavy workloads, complicated ADR reporting procedures, limitations of the e-health system, organizational culture, and lack of trust in the authority. Some of the perceived barriers were previously determined by other studies; however, novel barriers such as organizational culture and a lack of trust in authority have not been mentioned in previous research. The respondents also named a few facilitators; however, a better understanding of the barriers is necessary to design appropriate interventions and move the pharmacovigilance system in the right direction. Both GPs and CPs agreed that collaboration would positively affect patient care and expressed willingness to collaborate in the future. However, such a collaborative practice needs to be supported by complete and user-friendly guidelines.

## Figures and Tables

**Table 1:** Demographics of the participants.

Characteristics	Frequency
Gender	
Male	4
Female	8
Education	
Pharmacist	6
General Practitioner	6
Experience (Years)	
0–5	3
5–10	3
10–20	5
>20	1
ADR Reporting	
Yes	4
No	8

**Table 2:** Description of themes and subthemes.

Category	Themes and Subthemes
Knowledge	Theme 1: General knowledge about the definitions of pharmacovigilance and ADR Theme 2: Updating knowledge on pharmacovigilance
Practice	Theme 3: Practices related to ADR reporting
Barriers	Theme 4: Barriers to ADR reporting Subtheme 1: Lack of information and education Subtheme 2: Heavy workload Subtheme 3: Complicated ADR reporting procedure Subtheme 4: Limitations of the e-health system Subtheme 5: Organizational culture Subtheme 6: Lack of trust in authority
Facilitators	Theme 5: Facilitators of ADR reporting Subtheme 1: Improvement of the system Subtheme 2: Active support Subtheme 3: ADR map
Collaboration	Theme 6: Collaboration between GPs and CPs Subtheme 1: Importance of cooperation Subtheme 2: Need for a clear collaboration algorithm

**Table 3:** Identified barriers and suggested interventions.

Identified Barriers	Suggested Interventions [28,32]
Lack of information and education	Educational sessions: theoretical and practical
Heavy workload	Time management training Establishment of drug safety department/staff Computerized registration
Complicated ADR reporting procedure	Educational sessions: theoretical and practical Computerized registration Revision and reorganization of the reporting procedure
Limitations of the e-health system	Computerized registration Improvement of e-health system
Organizational culture	Organization of culture influence Educational sessions: theoretical and practical
Lack of trust in authority	Provision of active feedback and support Initiation of open discussions Educational sessions: theoretical and practical

## Data Availability

The data presented in this study are available on request from the corresponding author. The data are not publicly available due to confidentiality policies.
